# LINC01272/miR-876/ITGB2 axis facilitates the metastasis of colorectal cancer via epithelial-mesenchymal transition

**DOI:** 10.7150/jca.55666

**Published:** 2021-05-05

**Authors:** Zhenqiang Sun, Qin Dang, Zaoqu Liu, Bo Shao, Chen Chen, Yuying Guo, Zhuang Chen, Quanbo Zhou, Shengyun Hu, Jinbo Liu, Weitang Yuan

**Affiliations:** 1 Department of Colorectal Surgery, The First Affiliated Hospital of Zhengzhou University, Zhengzhou 450052, Henan, China.; 2 School of Life Sciences, Zhengzhou University, Zhengzhou 450001, Henan, China.; 3 Department of Interventional Radiology, The First Affiliated Hospital of Zhengzhou, University, Zhengzhou, 450052, Henan, China.; 4 Academy of Medical Sciences, Zhengzhou University, Zhengzhou 450052, Henan, China.

**Keywords:** colorectal cancer, long noncoding RNA, LINC01272, EMT, metastasis

## Abstract

**Background:** At the time of diagnosis, colorectal cancer (CRC) patients are usually in an advanced stage of disease, which is accompanied by metastasis. Long noncoding RNAs (lncRNAs) play critical regulatory roles in cancer biology. However, the contributions of lncRNA LINC01272 to CRC remain elusive.

**Methods:** Bioinformatics and the survminer R package were used to predict intermolecular correlations and prognostic indicators. Quantitative real-time PCR was used to examine molecular expression. *In vitro* experiments, including migration assays, invasion assays, and wound healing assays, were used to investigate the effects of LINC01272, ITGB2 and miR-876 on CRC cell migration and invasion abilities. Furthermore, a dual-luciferase reporter gene assay was performed to explore the potential mechanism by which LINC01272 contributes to CRC.

**Results:** We found that LINC01272 was highly expressed in multiple cancers and closely related to core epithelial-mesenchymal transition (EMT) factors and that high levels of LINC01272 are associated with a poor prognosis in CRC patients. qRT-PCR revealed that LINC01272 was highly expressed and negatively associated with miR-876 in CRC. Additionally, LINC01272 or ITGB2 silencing reduced, while miR-876 overexpression promoted, the invasiveness and metastatic capacity of CRC cells *in vitro*. Moreover, LINC01272 potentially targeted miR-876, and miR-876 potentially targeted ITGB2.

**Conclusion:** LINC01272 was highly expressed in CRC and predicted a poor prognosis. LINC01272 promoted EMT and metastasis by regulating miR-876/ITGB2 to act as an oncogene in CRC. LINC01272 may be a promising prognostic biomarker and therapeutic target for the treatment of CRC patients in the future.

## Introduction

Colorectal cancer (CRC) is the third most common type of cancer and the third leading cause of cancer-related death worldwide 1. The overall 5-year survival rate of patients with CRC is 65%. Approximately 12% of individuals under 50 years of age will be newly diagnosed with CRC 2. Therefore, robust diagnostic, prognostic and predictive biomarkers are clearly and urgently needed to detect advanced colon polyps and early-stage CRC, which are the forms of CRC that are most effectively treated with the current therapies available to specific CRC patients. The last two decades of research have demonstrated the potential of long noncoding RNAs to be used as biomarkers of CRC 3, 4. Continued investigation of this promising class of biomarkers is likely to lead to high-efficiency assays that can be used to prevent and manage CRC in patients 5. LncRNAs are a class of RNA molecules defined as transcripts longer than 200 nucleotides that lack protein coding potential 6. Increasing evidence suggests that lncRNAs may mediate oncogenic or anti-oncogenic effects and may be a new class of cancer biomarkers and therapeutic targets 7-9. Haberman Y et al. reported that LINC01272 upregulation was significantly associated with mucosal damage in early-onset Crohn's disease (CD) 10. In CD, upregulated LINC01272 was associated with a myeloid proinflammatory signature and regulated this signature in response to inflammatory signals. LINC01272 was also reported to be significantly upregulated in tissue and plasma samples from patients with inflammatory bowel disease (IBD) compared with those from healthy controls. Correspondingly, LINC01272 was considered a potential novel diagnostic biomarker of IBD 11. LINC01272 is expected to be used as a potential diagnostic biomarker of gastric cancer that acts by regulating the expression of EMT-related proteins to promote the metastatic ability of gastric cancer cells 12. Nevertheless, the role of LINC01272 in CRC remains elusive.

Therefore, we aimed to further explore the effect of LINC01272 in order to assess whether LINC01272 can become a useful diagnostic and prognostic biomarker and an important therapeutic target for the treatment of CRC patients. The expression patterns of LINC01272 in CRC cancer tissues were analysed, and its biological function in colon cancer cells was investigated to reveal a tumour biomarker and therapeutic target gene in CRC.

## Materials and methods

### Tissue specimen and clinical data collection

This study was approved by the First Affiliated Hospital of Zhengzhou University. A total of 27 paired CRC tissues and matched adjacent non-tumour tissues were obtained from patients who underwent surgical resection at The First Affiliated Hospital of Zhengzhou University. None of the patients received any preoperative chemotherapy or radiotherapy. Written informed consent was obtained from all the patients. The inclusion criteria were as follows: no preoperative chemotherapy, radiotherapy, or targeted therapy; no other types of tumours; and no autoimmune diseases. The specimens obtained during surgery were immediately snap frozen in liquid nitrogen and stored at -80 °C until RNA extraction. Clinical staging of the specimens was based on the NCCN (2019) guidelines.

### Bioinformatic prediction

RNA-Seq (FPKM normalized) data were retrieved from the TCGA portal, the Kaplan-Meier method was used to assess the prognostic significance of LINC01272, and the optimal cut-off value was determined by the survminer R package (Figure S1). The RNA-Seq datasets in GEPIA (http://gepia.cancer-pku.cn/) were used to analyse the expression and prognostic relevance of LINC01272, ITGB2, and EMT core factors. The potential role of LINC01272 in targeting miR-876 was predicted using DIANA tools (http://carolina.imis.athena-innovation.gr/diana_tools/). The potential role of miR-876 in targeting ITGB2 was predicted through the miRbase, miRecords and TargetScan databases.

### RNA extraction and reverse transcription

Total RNA was isolated from CRC tissues, paired adjacent non-cancerous tissues and CRC cells with RNAiso Plus reagent (Takara, Dalian, China), according to the manufacturer's instructions. The RNA quality was evaluated using a NanoDrop One C (Waltham, MA, USA), and the RNA integrity was assessed using agarose gel electrophoresis. An aliquot of 1 µg of total RNA was reverse-transcribed into complementary DNA (cDNA) using a High-capacity cDNA Reverse Transcription kit (TaKaRa Bio, Japan), according to the manufacturer's protocol. miRNAs were reverse transcribed using a miRNA reverse transcription kit (TaKaRa Bio, Japan).

### Quantitative real-time PCR

Quantitative real-time PCR (qRT-PCR) was performed using SYBR Assay I Low ROX (Eurogentec, USA) and SYBR® Green PCR Master Mix (Yeason, Shanghai, China) to detect gene expression. The 2^-ΔΔCt^ method was used to calculate the relative levels of gene and miRNA expression. The primers are listed in Table S1. GAPDH or U6 was used as the endogenous control for normalization. qRT-PCR assays were performed in triplicate with the following conditions: (1) 95 °C for 5 min and (2) 40 cycles of 95 °C for 10 s and 60 °C for 30 s. The relative expression of LINC01272 was calculated using the ΔCT (Ct lncRNA-Ct GAPDH) method. miRNA qPCR was carried out according to a miRNA qPCR kit (TaKaRa Bio, Japan), and U6 was used as the internal reference.

### Cell culture

The HCT116 human colon cancer cell line was purchased from the Shanghai Institute of Cell Biology, Chinese Academy of Sciences (Shanghai, China). The HCT116 cells were cultured in high-glucose DMEM (Sigma, D6429) containing 10% FBS (Biological Industries, Kibbutz Beit Haemek, Israel) and incubated at 37 °C in 5% CO2.

### siRNA transfection of colon cancer cells

Silencer select small interfering RNAs (siRNAs) specific for LINC01272 and a control siRNA were obtained from RiboBio (Guangzhou, China). To silence RNA molecules in colon cancer cells, LINC01272-specific siRNA (si-h-LINC01272, target sequence: GGAGAATAAACCCTCGGAT), ITGB2-specific siRNA (si-h-ITGB2, target sequence: GTACAAGAGGAGCAACGAA) and control siRNA were transfected into HCT116 cells. The transfection was conducted with Lipofectamine 3000 (Thermo Fisher, L3000-015), according to the manufacturer's instructions.

### Invasion and migration assays

To assess the migratory ability of the transfected cells, the cells were seeded in 24-well plates (Corning, NY, USA) and incubated under permissive conditions until they reached 90% confluence. After serum starvation for 24 h, wounds were created in the confluent cells with a 20-μL pipette tip. Then, the scraped cells were lightly washed with PBS, and serum-free medium was added to the 24-well plates. Wound healing within the scrape was then observed and photographed at the indicated time points, and the area was analysed by ImageJ (version 1.30, free at http://rsb.info.nih.gov/ij/).

According to the experimental objective of analysing invasion or migration, Transwell chambers (Corning, NY, USA) were prepared with or without Matrigel. Then, blood serum medium (10% FBS) was added to the lower chamber. After transfection with si-LINC01272 and inhibitor control, HCT116 cells were digested to prepare a cell suspension, and this suspension was added to the upper chamber and incubated for 24 h. At the end of this incubation, the residual cells in the upper chamber were gently wiped with cotton swabs. The cells were fixed with 4% paraformaldehyde and stained with 1% crystal violet for 30 min. After washing three times with PBS, the cells were imaged and counted with an OLYMPUS FV1000 confocal microscope. Every experiment was performed three times for statistical analysis.

### Dual-luciferase reporter assay

Luciferase activity assays were performed with the Dual-Luciferase Reporter Assay System (Promega, Madison, WI, USA). Putative wild-type (WT) and mutant (Mut) miR-876-5p-binding sites in the 3ʹ-UTR of LINC01272 or ITGB2 mRNA, termed LINC01272-Luc WT or LINC01272-Luc MT and ITGB2-Luc WT or ITGB2-Luc MT, were cloned into the psiCHECK2 vector (Promega, Madison, WI, USA). HCT116 cells were transfected with miR-876 mimics, the Renilla luciferase reporter vector (Promega), and the LINC01272-Luc WT, LINC01272-Luc MT, ITGB2-Luc WT or ITGB2-Luc MT reporter constructs using Lipofectamine 3000 reagent (Thermo Fisher, L3000-015), according to the manufacturer's instructions. As the internal control, 5 ng/well Renilla luciferase plasmid was used. After 48 h of transfection, the firefly and Renilla luciferase activities were measured using the Dual-Luciferase Reporter Assay System. Correction for differences in transfection efficiency was performed by normalizing the firefly luciferase activity to the total Renilla luciferase activity.

### Statistical analyses

All the statistical analyses were performed using SPSS version 24.0 (SPSS, Chicago, IL, USA) and GraphPad Prism 5.0 software (CA, USA). All the measurements were conducted in triplicate. Significant differences between the LINC01272 expression levels were analysed using two-sided Student's t-test. The indicated P < 0.05 was considered statistically significant.

## Results

### LINC01272 was highly expressed in colorectal cancer

**Figure 1:**
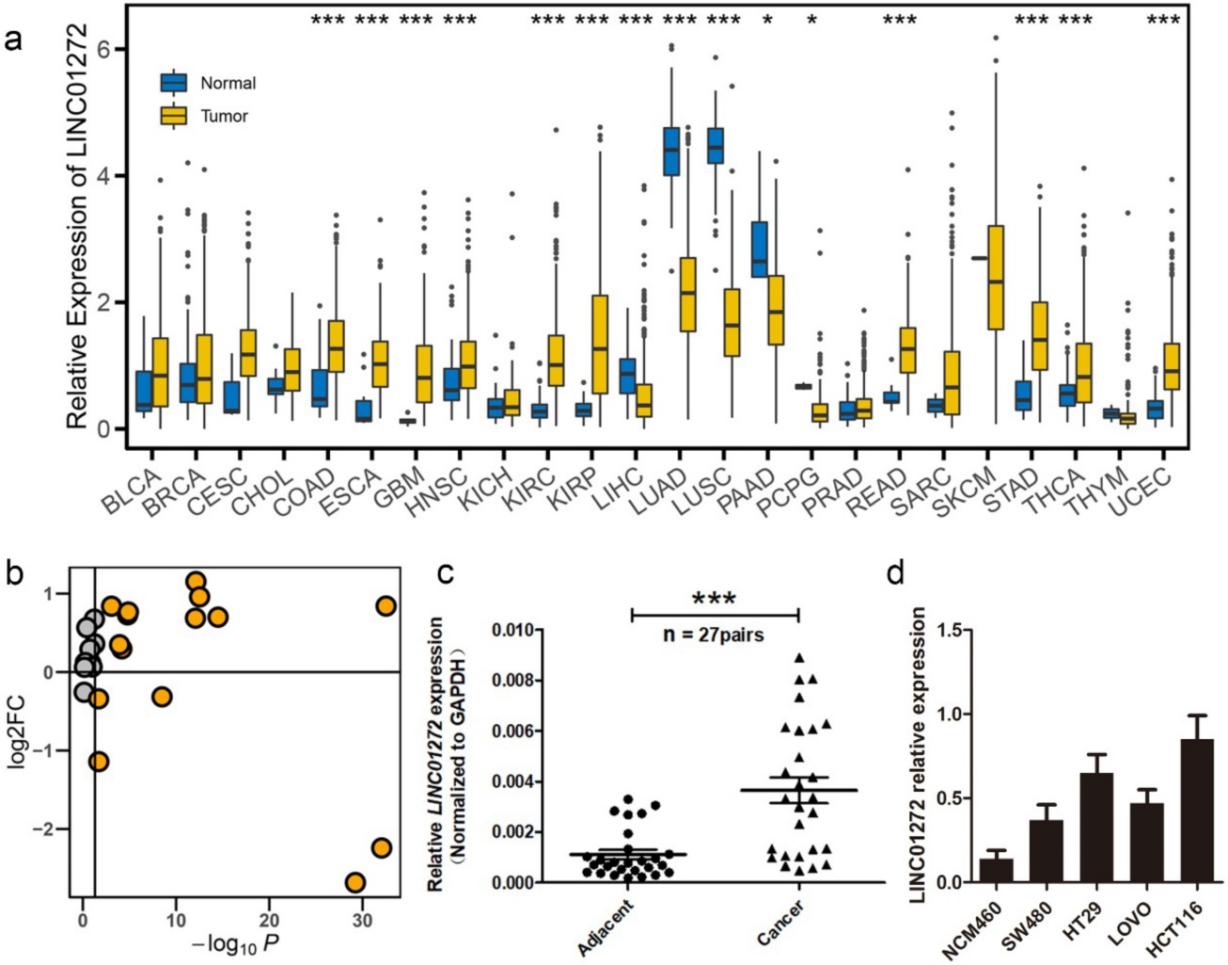
** LINC01272 was highly expressed in CRC. a,** LINC01272 expression in the normal and tumour groups in pan-cancer analysis. The expression data were retrieved from the UCSC portal (*P < 0.05, **P < 0.01 and *** P < 0.001). **b,** The log2FC and P-value distribution of LINC01272 between the normal and tumour groups in pan-cancer analysis. LINC01272 was upregulated in a variety of tumours, and in 10 of these tumours, the upregulation was significant. **c,** In 27 paired human colon cancer tissues and adjacent non-cancerous tissues, LINC01272 was more highly expressed in tumour tissues than in normal adjacent normal tissues (P<0.001). **d,** LINC01272 showed higher expression in multiple CRC cells (SW480, HT29, LOVO, and HCT116) than in normal intestinal mucosa cells (NCM460).

We first examined the expression of LINC01272 in the normal and tumour groups via pan-cancer analysis. The expression data were retrieved from the UCSC portal. The analysis results showed that LINC0172 was highly expressed in multiple cancers, including colon adenocarcinoma (COAD), oesophageal carcinoma (ESCA), glioblastoma multiforme (GBM), head and neck squamous cell carcinoma (HNSC), kidney renal clear cell carcinoma (KIRC), kidney renal papillary cell carcinoma (KIRP), liver hepatocellular carcinoma (LIHC), lung adenocarcinoma (LUAD), lung squamous cell carcinoma (LUSC), pancreatic adenocarcinoma (PAAD), pheochromocytoma and paraganglioma (PCPG), rectum adenocarcinoma (READ), stomach adenocarcinoma (STAD), thyroid carcinoma (THCA) and uterine corpus endometrial carcinoma (UCEC) (Figure 1a, b). In addition, we further analysed the expression of LINC01272 in 27 pairs of CRC tissues and adjacent normal mucosal tissues using qRT-PCR, and the results showed that LINC01272 was prominently overexpressed in colorectal cancer (P < 0.001) (Figure 1c), indicating that LINC01272 may facilitate CRC carcinogenesis. We also explored LINC01272 expression in different CRC cells and found that LINC01272 was highly expressed in SW480, HT29, LOVO and HCT116 cells compared with normal intestinal mucosa cells (NCM460). LINC01272 was the most highly expressed in HCT116 cells, which were the cells most easily transfected with plasmids (Figure 1d). Furthermore, we performed the following *in vitro* assays in HCT116 cells.

### High expression of LINC01272 predicted poor prognosis of CRC patients

**Figure 2:**
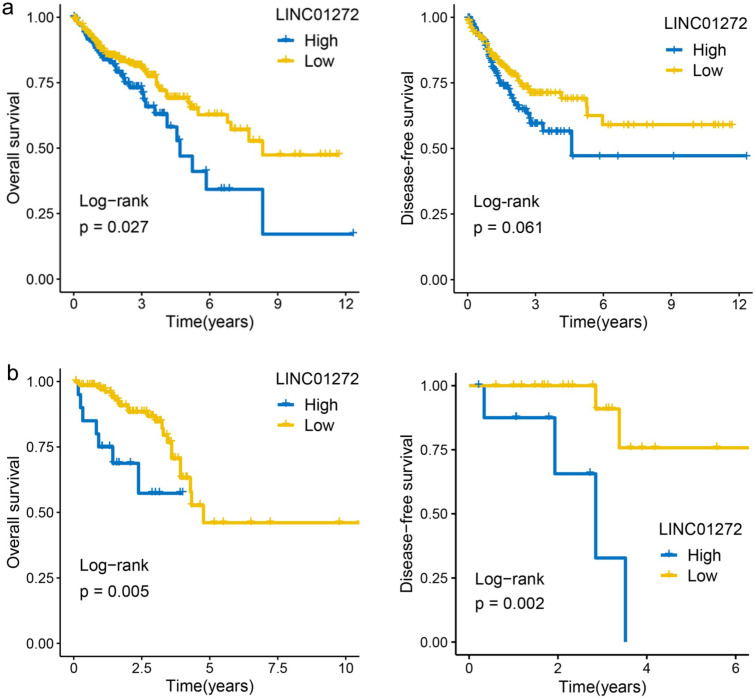
** High expression of LINC01272 predicts poor prognosis of CRC patients. a,** The OS rate of colon cancer patients with high LINC01272 expression was higher than that of patients with low LINC01272 expression (P<0.05), and the DFS showed valuable trends. **b,** The OS and DFS rates of rectal cancer patients of high LINC01272 expression were higher than those of rectal cancer patients with low LINC01272 expression (P<0.05).

The prognostic value of lncRNA expression was evaluated by the survminer R package and showed that the overall survival (OS) rate of COAD patients with high LINC01272 expression was higher than that of COAD patients with low LINC01272 expression (122 patients, P<0.05) (Figure 2a). The OS and disease-free survival (DFS) rates of READ patients with high LINC01272 expression were higher than those of READ patients with low LINC01272 expression (32 patients, P<0.05) (Figure 2b). Therefore, LINC01272 has promising prognostic value in patients with CRC.

### Both LINC01272 and ITGB2 were closely associated with core EMT molecules in CRC

**Figure 3:**
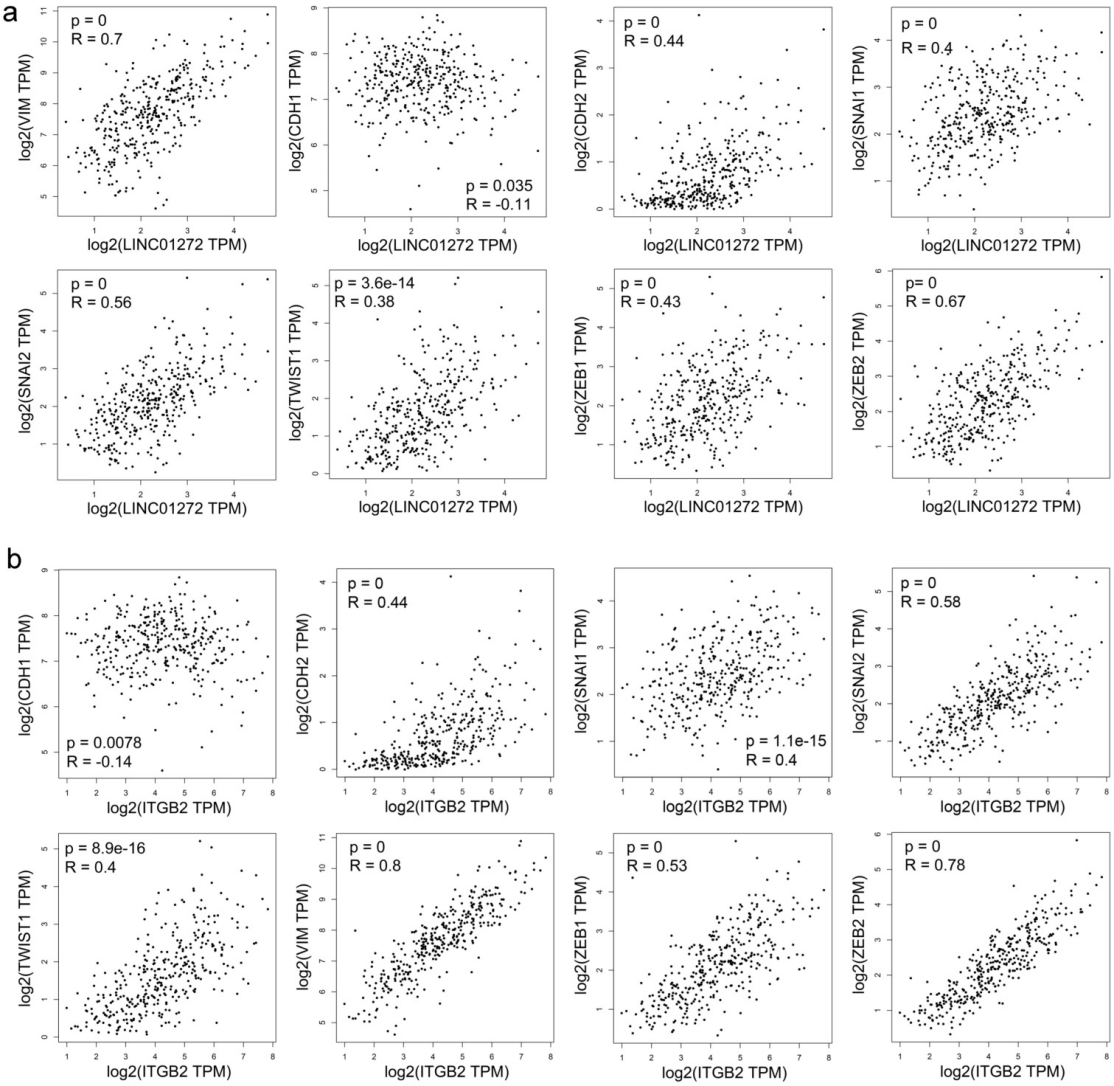
** Both LINC01272 and ITGB2 were closely associated with core EMT molecules in CRC. a,** LINC01272 expression was positively associated with VIM (Vimentin), CDH2 (N-cadherin), SNAI2 (SLUG), SNAI1 (SNAI), TWIST1 (TWIST), ZEB1, and ZEB2 (all P<0.001) and negatively associated with CDH1 (E-cadherin) (P<0.05). **b,** ITGB2 expression was positively associated with VIM (Vimentin), CDH2 (N-cadherin), SNAI2 (SLUG), SNAI1 (SNAI), TWIST1 (TWIST), ZEB1, and ZEB2 (all P<0.001) and negatively associated with CDH1 (E-cadherin) (P<0.01).

Correlation analysis was carried out with the RNA-Seq GEPIA dataset. The results demonstrated that LINC01272 expression was positively associated with VIM (Vimentin), CDH2 (N-cadherin), SNAI2 (SLUG), SNAI1 (SNAI), TWIST1 (TWIST), ZEB1, and ZEB2 (all P<0.001) and negatively associated with CDH1 (E-cadherin) (P<0.05) (Figure 3a). ITGB2 expression was positively associated with VIM (Vimentin), CDH2 (N-cadherin), SNAI2 (SLUG), SNAI1 (SNAI), TWIST1 (TWIST), ZEB1, and ZEB2 (all P<0.001) and negatively associated with CDH1 (E-cadherin) (P<0.01) (Figure 3b). Thus, both LINC01272 and ITGB2 were closely associated with EMT in CRC.

### LINC01272 was closely associated with ITGB2 in CRC

**Figure 4:**
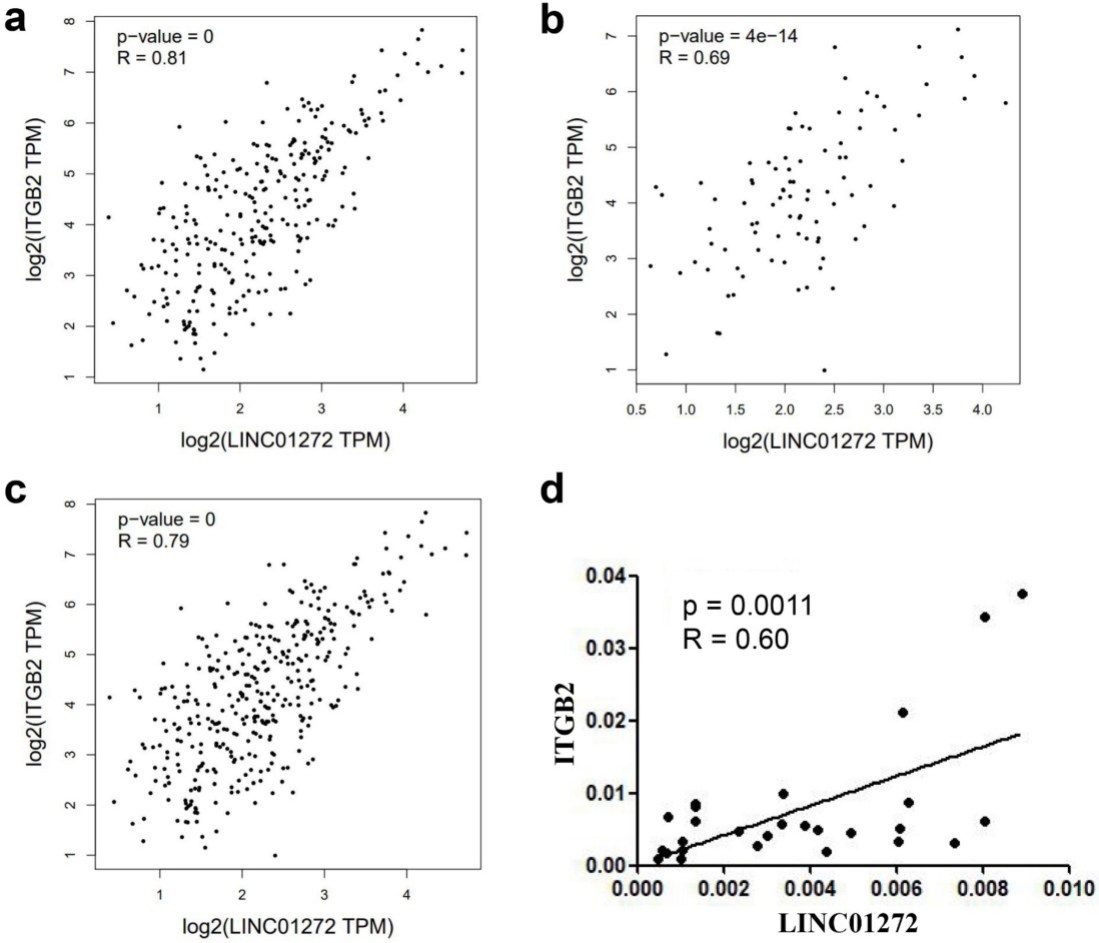
** LINC01272 was closely associated with ITGB2 in CRC. a, b and c,** LINC01272 was positively associated with ITGB2 in CRC based on analysis of the RNA-Seq dataset GEPIA (all P<0.001). **d,** In 27 paired human CRC tissues, LINC01272 was considerably positively associated with ITGB2, according to qRT-PCR assay (P<0.05).

The relationship between LINC01272 and ITGB2 was analysed with the RNA-Seq dataset GEPIA, and the results showed that LINC01272 was positively associated with ITGB2 in colon cancer, rectal cancer and CRC (P<0.001) (Figure 4a, b, c). Additionally, ITGB2 and LINC01272 were detected by qRT-PCR assay in 27 pairs of CRC samples. Correlation analysis was performed and showed that LINC01272 was positively associated with ITGB2 (P<0.05) (Figure 4d).

### LINC01272, miR-876 and ITGB2 regulated invasion and migration *in vitro*

**Figure 5:**
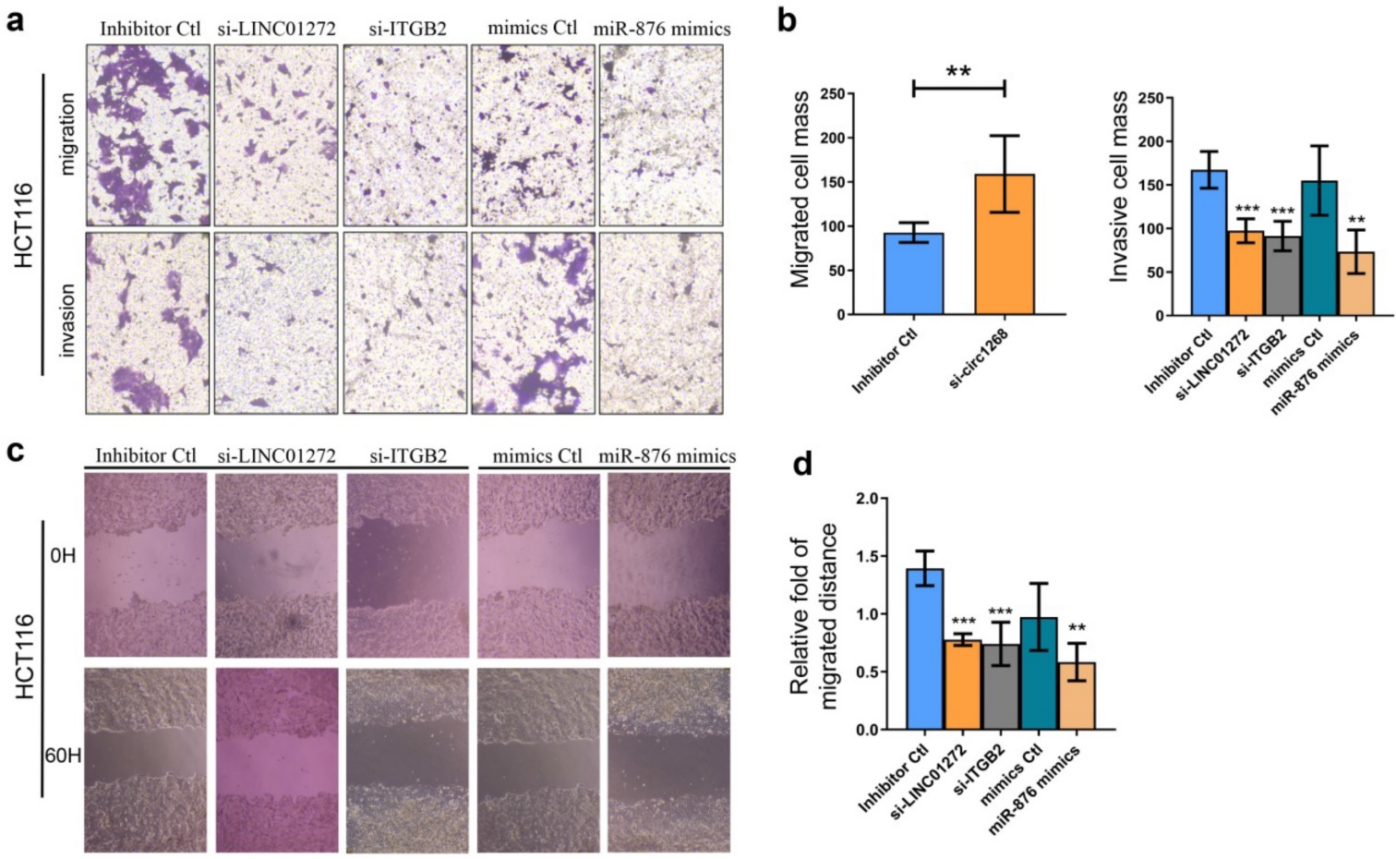
** LINC01272, miR-876 and ITGB2 regulated invasion and migration *in vitro*.** For the Transwell assay, HCT116 cells were transfected with si-LINC01272, si-ITGB2, miR-876 mimics and their corresponding controls. **a and b,** Inhibition of LINC01272 or ITGB2 expression or overexpression of miR-876 impaired the migration and invasion of HCT116 cells. **c and d,** Wound healing assay showed that down-regulation of LINC01272 or ITGB2 weakened the migration and motility of HCT116 cells. *p <0.05, **p < 0.01 and ***p < 0.001 compared to the control group by Student's t-test.

To explore the roles of LINC01272, miR-876 and ITGB2 in CRC cells, HCT116 cells were transfected with LINC01272 siRNA (si-LINC01272), ITGB2 siRNA (si-ITGB2), miR-876 mimics and their corresponding controls. The knockdown and overexpression efficiencies were confirmed via qRT-PCR analysis (Figure S2). Transwell assays were carried out and showed that by inhibiting LINC01272 or ITGB2 expression, miR-876 overexpression inhibited the migration and invasion of HCT116 cells (Figure 5a, b). Similarly, a wound healing assay further verified that by inhibiting LINC01272 or ITGB2 expression, miR-876 overexpression reduced the migration and motility of HCT116 cells (Figure 5c, d). Accordingly, LINC01272, miR-876 and ITGB2 could promote the invasion and migration of CRC cells.

### LINC01272 bound to miR-876 and miR-876 targeted ITGB2 in CRC

**Figure 6:**
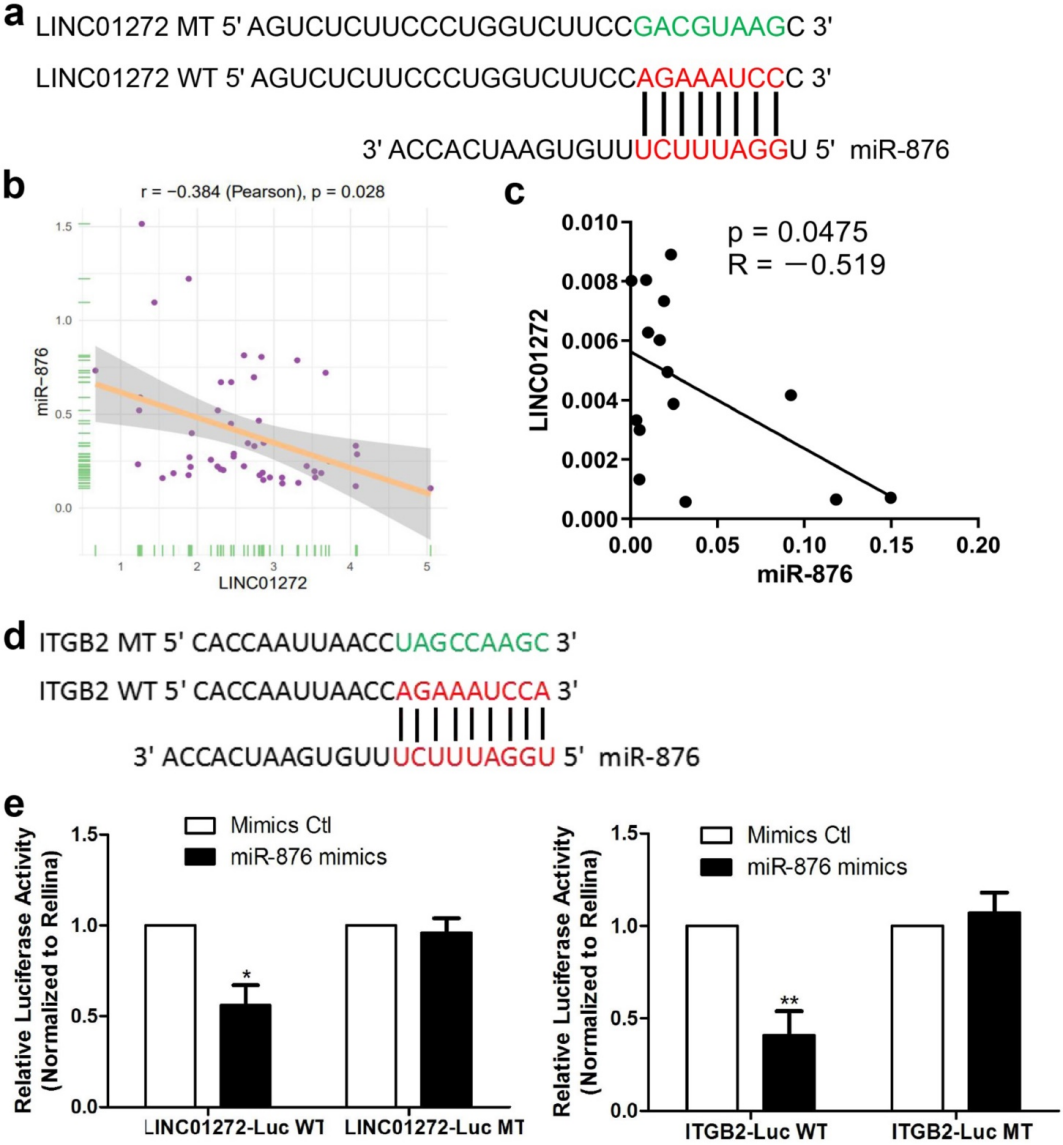
** LINC01272 bound to miR-876 and miR-876 targeted ITGB2 in CRC. a,** Prediction analysis with the DIANA lab database showed that LINC01272 contained a miR-876-binding sequence. Based on the above, a LINC01272-Luc WT plasmid and LINC01272-Luc MT plasmid were constructed. **b and c,** Bioinformatics and qRT-PCR analyses were performed to detect LINC01272 and miR-876 and showed that these two factors were negatively associated (P<0.05). **d,** Based on prediction analysis of the miRbase, miRecords and TargetScan databases, the ITGB2 3'UTR contained a miR-876-binding sequence. **e,** A LINC01272-Luc WT plasmid, LINC01272-Luc MT plasmid and their corresponding controls were transfected into HCT116 cells. Transwell assays were carried out and showed that miR-876 inhibited the luciferase activity of the LINC01272-Luc WT plasmid but not the luciferase activity of the LINC01272-Luc MT plasmid in HCT116 cells. Similarly, Transwell assays were performed and showed that miR-876 inhibited the luciferase activity of the ITGB2-Luc WT plasmid but not the luciferase activity of the ITGB2-Luc MT plasmid in HCT116 cells. Three independent experiments were performed. *p <0.05, **p < 0.01 compared to the control group by Student's t-test.

To study the mechanism underlying the effect of LINC01272, bioinformatic prediction was conducted via the DIANA lab database. The prediction analysis results showed that LINC01272 had a miR-876-binding sequence (Figure 6a). Based on the prediction results, a LINC01272-Luc WT plasmid and LINC01272-Luc MT plasmid were constructed. In addition, bioinformatic prediction with rstudio (version 1.0.136) and the R package revealed a negative correlation between LINC01272 and miR-876 in CRC (Figure 6b). qRT-PCR was performed to analyse 15 CRC samples. Correlation analysis showed that the LINC01272 expression levels were negatively associated with the miR-876 expression levels in CRC (P<0.05) (Figure 6c). The LINC01272-Luc WT plasmid, LINC01272-Luc MT plasmid and their corresponding controls were transfected into HCT116 cells. Transwell assays were carried out and showed that miR-876 inhibited the luciferase activity of the LINC01272-Luc WT plasmid but not the luciferase activity of the LINC01272-Luc MT plasmid in HCT116 cells (Figure 6e). Moreover, bioinformatic prediction via prediction analysis of the miRbase, miRecords and TargetScan databases showed that the ITGB2 3'UTR contained a miR-876-binding sequence (Figure 6d). A ITGB2-Luc WT plasmid and ITGB2-Luc MT plasmid were constructed. Similarly, Transwell assays were performed and showed that miR-876 inhibited the luciferase activity of the ITGB2-Luc WT plasmid but not the luciferase activity of the ITGB2-Luc MT plasmid in HCT116 cells (Figure 6e). Consequently, LINC01272 might promote ITGB2 expression by targeting miR-876 in CRC.

### LINC01272/miR-876/ITGB2 axis regulated EMT and metastasis

**Figure 7:**
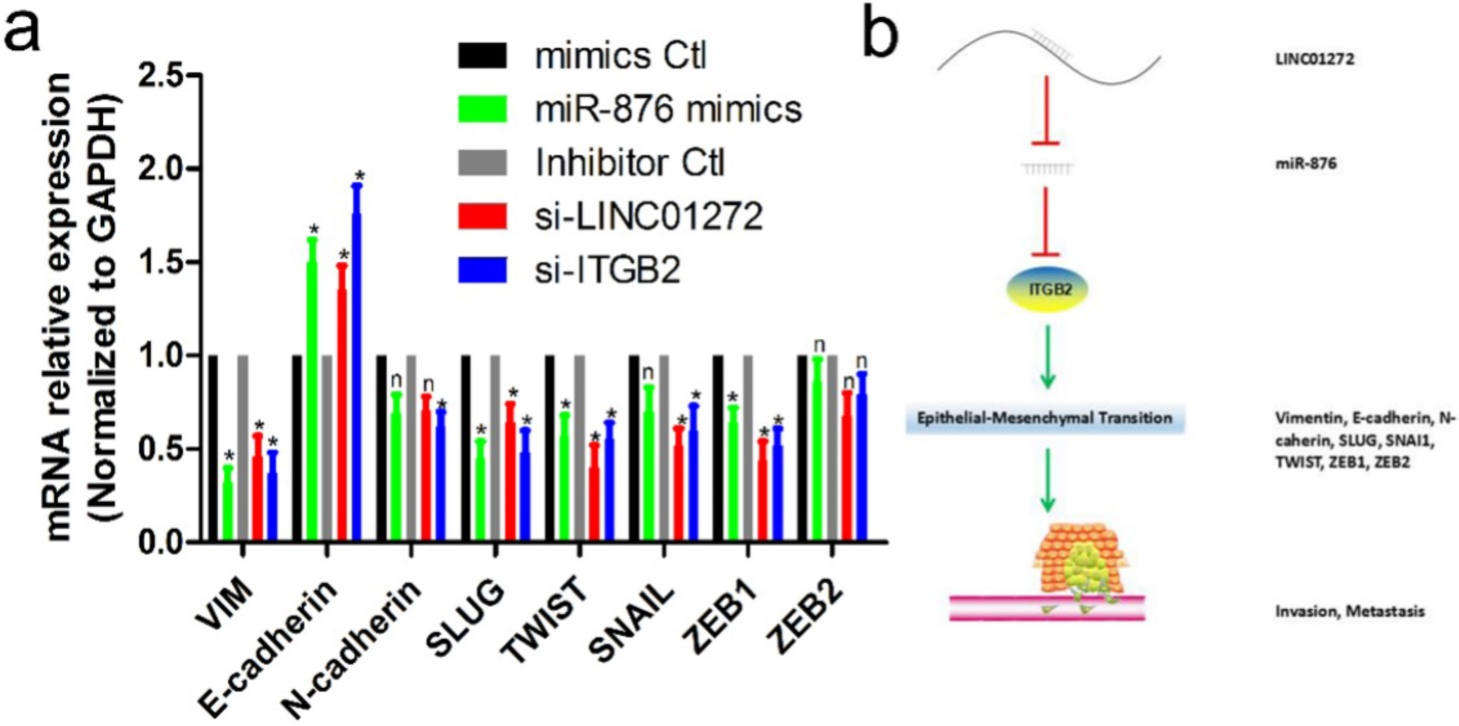
** The LINC01272/miR-876/ITGB2 axis regulated EMT and metastasis. a,** HCT116 cells were transfected with si-LINC01272, si-ITGB2, miR-876 mimics and their corresponding controls. After culturing for 48 hours, RNA was extracted for qRT-PCR assay. The results showed that by inhibiting LINC01272 or ITGB2 expression, miR-876 overexpression inhibited the expression of EMT core factors, including VIM, E-cadherin, SLUG, TWIST and ZEB1. **b,** Schematic representation of a model depicting the major molecular mechanisms of the LINC01272/miR-876/ITGB2 axis in CRC.

To verify the effects of LINC01272, miR-876 and ITGB2 on EMT in CRC, HCT116 cells were transfected with si-LINC01272, si-ITGB2, miR-876 mimics and their corresponding controls. After culturing for 48 hours, RNA was extracted for qRT-PCR assay. The results showed that by inhibiting LINC01272 or ITGB2 expression, miR-876 overexpression inhibited the expression of EMT core factors, including VIM, E-cadherin, SLUG, TWIST and ZEB1 (Figure 7a). In brief, LINC01272 enhanced the EMT process by regulating ITGB2-miR-876, consequently promoting the invasion and metastasis of CRC (Figure 7b).

## Discussion

CRC is the 3^rd^ most commonly diagnosed cancer in males and the 2^nd^ most commonly diagnosed cancer in females worldwide 13. Cancer metastasis is a process during which cancer cells disseminate from the primary tumour to distant organs. Additionally, the CRC patient population as a whole is rapidly shifting toward a younger age range 2. Therefore, the identification of novel biomarkers of advanced CRC is urgently needed to achieve early diagnosis and establish effective strategies with precise targets for improving patient prognosis.

Increasing studies have reported that lncRNAs have high tissue specificity and are expected to be promising drivers and biomarkers of various malignancies 14-18. LncRNAs derived from conserved genomic locations play crucial biological roles with conserved functionality during embryonic development 19. A large body of evidence has revealed the essential role of lncRNAs in all stages of angiogenesis and metastasis. For instance, we previously verified that downregulation of the long noncoding RNA ANRIL suppressed lymphangiogenesis and lymphatic metastasis in colorectal cancer 20. YAP1-induced MALAT1 could promote EMT and angiogenesis by sponging miR-126-5p in CRC 21. RP11 was aberrantly expressed at low levels in CRC, and its low expression was closely associated with aggressive clinicopathological features and unfavourable prognosis in CRC patients 22. Additionally, many previous studies have reported that lncRNAs could act as oncogenes or anti-oncogenes. The long noncoding RNA TTTY15, which is located on the Y chromosome, promoted prostate cancer progression by sponging let-7 23. LNMAT1 promoted the lymphatic metastasis of bladder cancer via CCL2-dependent macrophage recruitment 24. LncGata6 maintained the stemness of intestinal stem cells and promoted intestinal tumorigenesis 25. Downregulation of DGCR5 contributed to cervical cancer progression by activating Wnt signalling 26.

In our study, we first showed that LINC01272 was highly expressed in CRC tissues compared with normal colorectal tissues via an analysis of the GEPIA database. To the best of our knowledge, our study is the first to explore the biological effect of LINC01272 and demonstrate a significant association between LINC01272 and CRC prognosis. Further analysis showed that LINC01272 was closely associated with EMT-related molecules, such as VIM, CDH1, CDH2, SNAIL1, SNAI2, TWIST, ZEB1, and ZEB2. Extensive gene screening showed that ITGB2 had simultaneously negative correlations with these molecules. Currently, the role of ITGB2 remains unknown in CRC. In this study, ITGB2 was found to upregulate in CRC and positively correlate with LINC01272 expression. Our results also demonstrated that ITGB2 was involving in the proliferation, migration, and invasion of CRC. In order to further explore the potential relationship between LINC01272 and ITGB2, we performed bioinformatic predictions through the DIANA laboratory database. The results displayed that LINC01272 had binding sequences with miR-876. Further investigation revealed miR-876 displayed the inverse phenomenon relative to ITGB2. Thus, we hypothesized that a LINC01272/miR-876/ITGB2 axis might be involved in EMT process of CRC. Subsequently, dual-luciferase reporter assays confirmed that miR-876 had potential binding capacities with LINC01272 and ITGB2. Transwell and wound healing assays further verified that LINC01272/miR-876/ITGB2 axis could regulate invasion and migration of tumor cells. Overall, we reported that LINC01272/miR-876/ITGB2 axis might facilitate the metastasis of CRC via EMT process.

In conclusion, our study provides solid evidence that LINC01272 was highly expressed in CRC and that high LINC01272 expression predicted a poor prognosis. LINC01272 favoured EMT and metastasis by regulating miR-876/ITGB2 to act as an oncogene in CRC. LINC01272 will potentially become a prognostic biomarker and a therapeutic target for the treatment of CRC patients in the future.

## Supplementary Material

Supplementary figures and tables.

## Abbreviations

lncRNAs: Long noncoding RNAs
CRC: colorectal cancer
EMT: epithelial-mesenchymal transition
CD: Crohn's disease
IBD: inflammatory bowel disease
COAD: Colon adenocarcinoma
ESCA: Esophageal carcinoma
GBM: Glioblastoma multiforme
HNSC: Head and Neck squamous cell carcinoma
KIRC: Kidney renal clear cell carcinoma
KIRP: Kidney renal papillary cell carcinoma
LIHC: Liver hepatocellular carcinoma
LUAD: Lung adenocarcinoma
LUSC: Lung squamous cell carcinoma
PAAD: Pancreatic adenocarcinoma
PCPG: Pheochromocytoma and Paraganglioma
READ: Rectum adenocarcinoma
STAD: Stomach adenocarcinoma
THCA: Thyroid carcinoma
UCEC: Uterine Corpus Endometrial Carcinoma
OS: overall survival
DFS: disease free survival
