# Supplementary material for: LINC01272/miR-876/ITGB2 axis facilitates the metastasis of colorectal cancer via epithelial-mesenchymal transition
Source: J Cancer. 2021 May 5;12(13):3909–19. doi: 10.7150/jca.55666 (PMC8176243; doi:10.7150/jca.55666)
Supplement: Supplementary file 1 — Supplementary figures and tables. [file jcav12p3909s1.pdf]

Supplementary figure 1

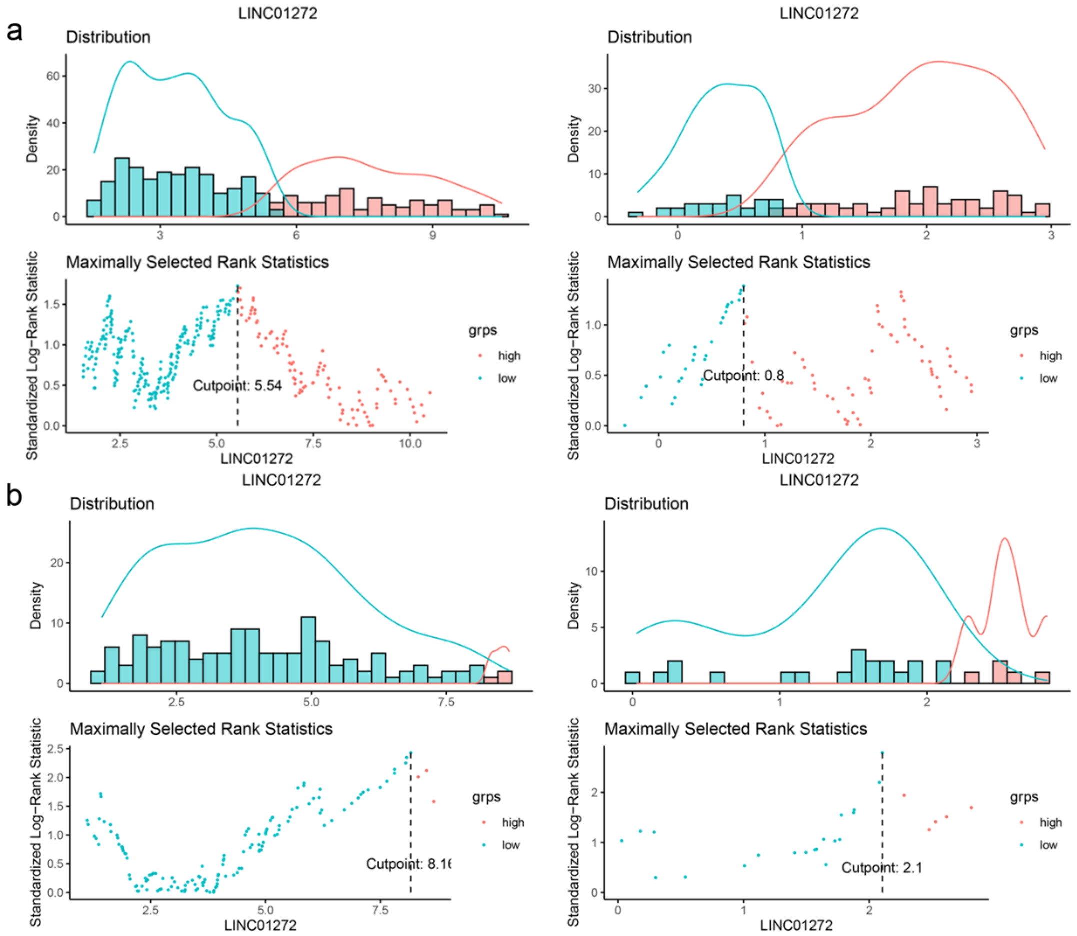

Figure S1 Selection of the optimal cutoff value. a. The optimal cutoff value of LINC01272 expression when analyzing the prognosis (OS rate and DFS rate) of COAD patients. b. The optimal cutoff value of LINC01272 expression when analyzing the prognosis (OS rate and DFS rate) of READ patients.

Supplementary figure 2

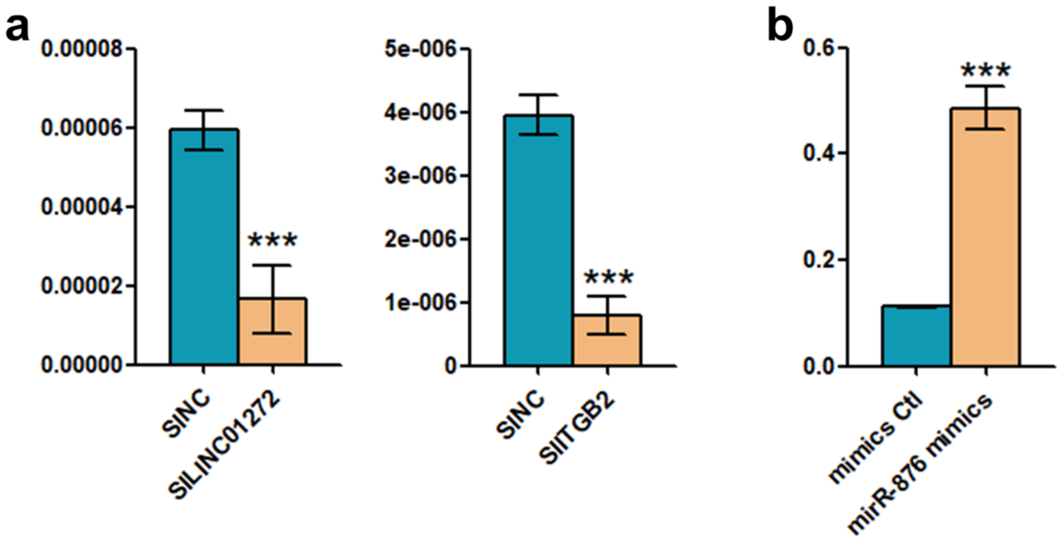

Figure S2 Cell transfection efficiency. a. The knockdown efficiency of LINC01272 and ITGB2. b. The overexpression efficiency of miR-876.

Supplementary figure 3

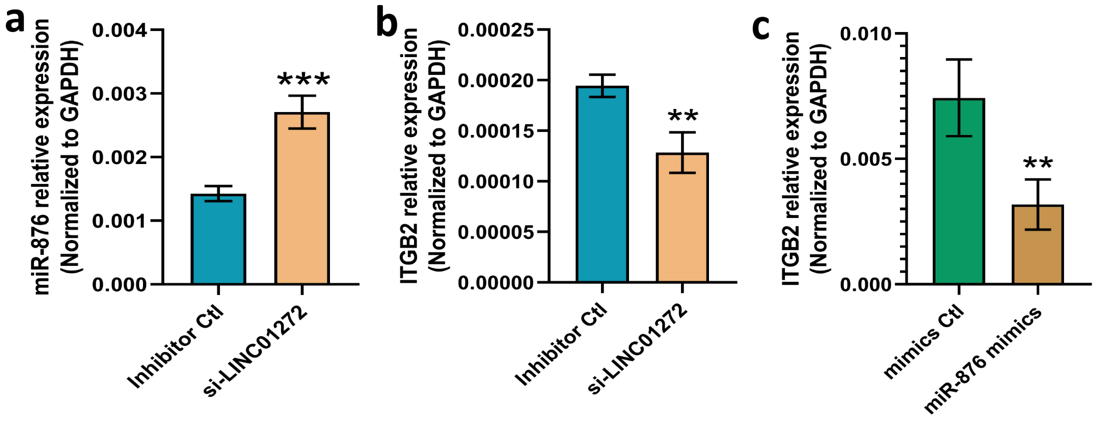

Figure S3 Expression of related molecules after knockdown of LINC01272 or overexpression of miR-876. After knockdown of LINC01272, miR-876 expression was increased (A) and ITGB2 expression was decreased (B). And the relative expression of ITGB2 was reduced upon overexpression of miR-876 (C).

Supplementary table

Table S1 The forward and reverse primers for qRT-PCR

Genes	Forward (5'- to 3'-)	Reverse (5'- to 3'-)
LINC01272	CCAGGACAAGTGCCCAGTAAAC	TGGTGGCAGTGGAGGCAGAA
hsa-miR-876-5p	CCGCGCGTGGATTTCTTTGTGAA	AGTGCAGGGTCCGAGGTATT
ITGB2	AGTGCAGGGTCCGAGGTATT	ACCAGAGCAGAGACCGCA
Vimentin	CAGTTTTTCCCAATGTAGCCA	AAGCAGGAGTCCACTGAGTACC
SLUG	TTCTCTTCCATTTCACGCATC	TCTTTGGGGCGAGTGAGTCC
TWIST	AGCTTCAATGGCATGGGGGT	CGGGAGTCCGCAGTCTTACG
SNAIL	GCTCAGCTTGTCCGAGGGCA	GGAAGCCTAACTACAGCGAGC
CDH1	GATGAGCATTGGCAGCGA	CTCTCGGTCCAGCCCAGTGGT
CDH2	TCGCCATCCAGACCGACCCA	GCAGTTGACTGAGGCGGGTGC
ZEB1	GCAGAUACUACACCAACUCTT	AUGGUGGUUAGUCAGUUGCTT
ZEB2	AACACCCCTGGCACAACAAC	GCTGACTGCATGACCATCGC
U6	GCTTCGGCAGCACATATACT	GTGCAGGGTCCGAGGTATTC
GAPDH	GGAGCGAGATCCCTCCAAAAT	GGCTGTTGTCATACTTCTCATGG
